# The Influence of the ITO Layers’ Thicknesses on Their Chosen Physical Surface Parameters

**DOI:** 10.3390/ma16041363

**Published:** 2023-02-06

**Authors:** Małgorzata Musztyfaga-Staszuk, Katarzyna Gawlińska-Nęcek, Robert Socha, Piotr Panek

**Affiliations:** 1 Welding Department, Silesian University of Technology, Konarskiego 18A, 44-100 Gliwice, Poland; 2 Institute of Metallurgy and Materials Science PAS, Reymonta 25, 30-059 Krakow, Poland; 3 Centrum Badań i Rozwoju Technologii dla Przemysłu S.A., Waryńskiego 3A, 00-645 Warszawa, Poland

**Keywords:** transparent conductive oxides, work function, indium tin oxide (ITO)

## Abstract

The paper presents the results concerning the influence of the thickness of the ITO and In_2_O_3_ layers deposited by the magnetron sputtering method on the physical parameters characterising their surface properties. The characterisation parameters were obtained by atomic force microscopy (AFM), X-ray photoelectron spectroscopy (XPS), and Kelvin probe. The increase in the layers’ thickness related to the time of their fabrication causes an increase in the surface roughness and the value of the work function, followed by a decrease in the concentration of elements and compounds in the near-surface area.

## 1. Introduction

One can find several definitions in the literature about contact potential, photo electricity, thermal emission of electrons, and band theory of electrical conductivity. It is possible to determine the work function of the other conductor by measuring the contact voltage, i.e., the voltage between the surface of the two conductors, knowing the work function for one of the conductors. The contact potential measurement can be performed in two ways [1,2,3,4,5,6,7]. 

The first one concerns the measurement of the contact value of the contact potential difference (CPD) with the reference electrode (RE) at the Kelvin probe stand and then the calculation of the work of the electron work function (Φ). Knowing the parameter Φ enables defining the Fermi level for the tested material (e.g., semiconductor). Concerning the material, it is also necessary to determine the type and condition of its surface. Such factors include temperature, type and thickness of the adsorbate, and ionisation of element atoms. The structure of the Kelvin Probe includes an electric circuit with a regulated voltage source connected, an ammeter, and a vibrating ER electrode above the test sample. A reference electrode (usually made of a golden mesh) is used [8] to eliminate the material oxidation process and change its work function. In work [8], this concept was defined as the value of the difference between the work function of the gold vibrating electrode RE and the work function of the tested sample divided by the elementary charge, where the electric capacity of sample C is equal to the Q charge accumulated on the sample (Table 1) [8].

The study used indium tin oxide (ITO), which is commonly known as n-type transparent conductive oxide (TCO). It combines good electrical conductivity with optical transparency in the visible range [12,13,14]. It is widely used as a high-work function electrode for optoelectronic applications such as solar cells or organic light-emitting diodes (OLED) [15,16]. Moreover, transparent conducting oxides (TCOs) are essential materials used in flat panel displays, photovoltaics, and electrochromic windows [17]. These papers [18,19,20,21,22,23,24] show the determination of the work function of commercially available and surface-modified ITO substrates using the Kelvin probe.

The issue of the contact potential difference (CPD) method with the use of a Kelvin probe for the application of thin layers of transparent conductive oxides (TCO) was analysed also based on data contained in the “Elsevier (Science Direct)”, Web of Science, and Scopus databases. Table 2 shows that the Elsevier database recorded the most significant number of published works for all the issues searched except for one. The WOS database took second place, and the Scopus database was third in terms of the analysed issues. Based on the available literature resources from these databases in the years 1968 to 2020 for the WOS base, 1960 to 2020 for the Scopus lilac, and 1998 to 2020 for the Elsevier base, it can be stated that this is a topic that scientists have been interested in for a long time (state of knowledge as of 28 March 2022).

Table 3 summarises the results of specific issues in two WOS and Scopus databases from 2020. It can be concluded that a more significant number of published works related to issues related to background oxides are contained in the WOS database than in Scopus (the state of knowledge as of 4 May 2022). Analysing the obtained research results since information technology’s development, the creation of bibliographic lists has been moved to the virtual space, thanks to which the considered issues can be presented statistically. Databases help an increasing number of emerging scientific journals, which separate valuable scientific publications from those of poor quality of those that are unscientific.

The investigation is based on experimental results of the In_2_O_3_ and ITO thin film samples, usually polycrystalline, highly defective layers produced by magnetron sputtering. To probe the potential of In_2_O_3_ and ITO for contact layers in thin solar cells based on metal oxides (e.g., CuO), which is the future aim of our works, it is necessary to investigate the electronic structure and properties of both thin layers. This work is a series of publications on this topic. Obtaining a ready electronic structure with the highest efficiency value is possible by the mutual correlation of parameters with layers created in the optimized technological process of its manufacturing. The mentioned correlation is important in the technology of their products because various processes take place during their manufacture, which can cause both an increase and a decrease in the desired external parameters of the electronic structure. The decline may be due to the degradation of the physical parameters of the layers of the electronic structure. It belongs to the decisive following parameters [25]: electromagnetic radiation reflection coefficient, solar cell base material thickness, charge carrier concentration and its mobility, charge carrier effective lifetime, carrier diffusion path length charge, surface recombination speed, electromagnetic radiation absorption coefficient, spectral sensitivity, resistances of areas and structural elements of the solar cell, type, and concentration of defects and the resulting density of recombination centers. Electrical optical, and structural properties are integral measurements of the whole material. This paper does not present the results concerning the examination of the optical properties of the discussed materials as they were included in earlier papers [26,27].

## 2. Materials for Investigation

The magnetron sputtering method was applied to the deposition ITO and In_2_O_3_ layers. A constant current value (100 W) determining the set power was used for the samples. The thickness of the applied layers was also changed from 15 to 135 nm. The layering rate was monitored with a quartz microbalance. Into the investigation were applied p-type Cz-Si wafers ((100) orientation) with a thickness of 380 μm ± 25 μm and resistivity of 7.5–8 Ωcm. Borosilicate optical glasses and two ITO targets, suitable for producing mixed indium tin oxide (ITO) layers with a composition-dependent resistivity, were used for the tests. In this work, the following alloy composition was applied: In and Sn alloy type, 99.99% purity and 99:10 (wt.%). Preparation of samples for investigation included: silicon wafers, which were subjected to chemical etching processes, and optical glasses, which were chemically washed in an ultrasonic cleaner.

## 3. Methodology

Measurement of the contact value of the contact potential difference (CPD) investigated samples concerning the reference electrode (RE) (for gold of 4.815 eV) was performed on a Kelvin probe, and then work function (Φ) was calculated. The thickness of thin layers was measured at the Sentech SE 800PV spectral ellipsometer(Berlin, Germany) based on the Step Scan Analyzer measurement mode. Measurements were in the range of 240–980 nm, and the angles of incidence were 50, 60, and 70 degrees. To study the morphology of the investigated samples (the roughness) a Park System XE-100 (Suwon, Korea) atomic force microscope was used. A non-contact mode was applied with a probe elastic constant of 40 N/m and a resonance frequency of 300 kHz. The investigations were performed in the area of 10 × 10 and 1 × 1 micrometres. The X-ray photoelectron spectra (XPS) were acquired using hemispherical analyser EA 15 (PREVAC) equipped with dual anode X-ray source RS 40B1 (PREVAC, Brzeziny, Poland). The measurements were performed using Al Kα (1486.6 eV) radiation and an analyser pass energy of 100 eV. The spectra were recorded in normal emission geometry with an energy resolution of 0.9 eV. The binding energy scale of the analyzer was calibrated concerning Au 4f7/2 (84.0 eV) region of the gold-covered sample placed at the same sample stage [28]. The ultra-high vacuum (UHV) conditions of 8 × 10^−10^ mbar were maintained during the measurements. The analysis area was approximately 3 mm^2^, and the depth of analysis was about 10 nm. The spectra were analysed with the use of CasaXPS 2.3.24 software(Casa Software Ltd, Devon, United Kingdom). The electron binding energy (BE) scale was calibrated for the Fermi edge at 0.0 eV. The Shirley-type spectrum background was used.

Spectra were fitted using CASA XPS^®^ software with the use of embedded algorithms. If not specified, the components were fitted with a product of Gauss (30%) and Lorenz (70%). The spectra were compared to the background level.

## 4. Results and Discussion

Kelvin probe work function and thickness analysis were used. Using a Kelvin probe, the CPD was measured for the thin layers of In_2_O_3_ and ITO deposited by the magnetron sputtering technique on glass substrates. Each sample was measured in several places. Table 4 presents the Φ results of the obtained layers.

The work function for each sample was calculated based on the investigation results. For the indium oxide layer, no significant differences were observed in the calculated value of *ϕ* for different thicknesses. The experimentally determined work function for deposited In_2_O_3_ is about 4.6 eV, while the literature value is about 5 eV [29]. In the case of indium-doped tin oxide, the *ϕ* values seem to depend to some extent on the thickness of the layer. The highest work function among the measured oxides was shown by indium tin oxide for the thickest layers (70 and 100 nm), while indium oxide showed the lowest value for the layer thickness of 20 nm. Higher work function values of ITO layers compared to the value of the reference sample result from network defects. Thin layers are characterised by a lower work function value (4.33 eV for 25 nm and 4.37 eV for 45 nm), which may result from their surface composition and/or defects. Thicker layers (especially 135 nm) are characterised by the work function close to the theoretical value of 4.7–4.8 eV. The work function of the commercial ITO (Ossilla-product Code S111) reference sample was 4.85 eV.

**Figure 1:**
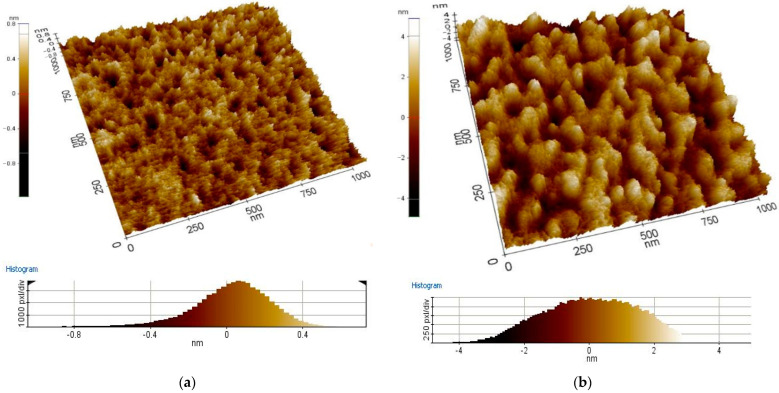
Topography of the applied In_2_O_3_ layers with thickness (**a**) 25 nm, (**b**) 135 nm (chosen example) (AFM).

**Figure 2:**
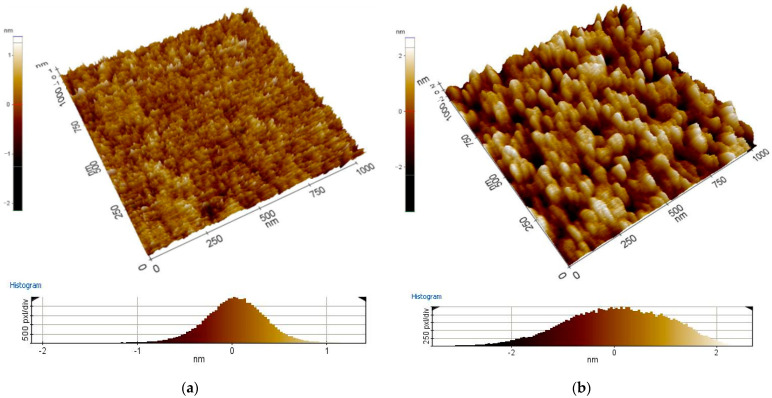
Topography of the applied ITO layers with thickness (**a**) 25 nm, (**b**) 135 nm (chosen example) (AFM).

AFM analysis is considered next. The topography of the deposited layers was investigated by AFM and presented in Figure 1 and Figure 2. Based on obtained In_2_O_3_ and ITO results, it was found that the layers with the most negligible thickness have a finer-crystalline structure than the layers with the most significant thickness. The results showed that ITO and In_2_O_3_ thin layers have a crystalline structure with a domain that increases in size with increasing thickness. Grown layers’ completeness depends on the quality of interfaces, which in turn depends on the number of properties, such as crystal structure and defects existing in a thin layer. Parameters, such as thickness, doping type, level, and other deposited conditions, should be optimized to optimize electrical parameters—for instance, the potential of the contact layer, low electrical resistance, as well optical parameters such as high transparency [30]. The roughness measurements showed an increase in In_2_O_3_ and ITO roughness with the layer thickness (Table 4).

XPS analysis is next considered. The surface composition and electronic states of the elements were analyzed for the fabricated samples. The acquired spectra were deconvoluted, taking into account the lowest number of components possible, the full width at half maximum (FWHM) of the analyzed peaks related to physically possible values for the given elements, the analyser resolution, and the chemical composition of the samples.

**Figure 3:**
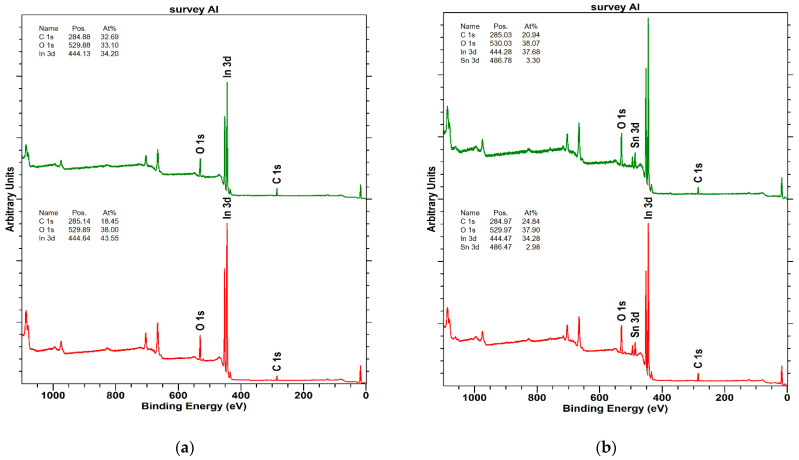
Survey spectra of (**a**) In_2_O_3_, (**b**) ITO sample surfaces (where: at the bottom −25 nm, at the top 135 nm).

Results for In_2_O_3_ and ITO are next considered. The results of In_2_O_3_ and ITO surface composition analysis on a basis of survey spectra are collected in Table 5 and Figure 3. The performed analysis shows that In_2_O_3_ layer of 135 nm is approximately twice more contaminated with the carbon species than the 25 nm sample. Such a result suggests that the In_2_O_3_ target is contaminated with carbon species, which are sputtered together with the main target component, i.e., In_2_O_3_. In case of ITO layers, the carbon contamination of the surfaces is independent of the deposition time. Moreover, the oxygen concentration remains constant for the same deposition conditions, which confirms that the sputtering targets of ITO and In_2_O_3_ are different.

**Figure 4:**
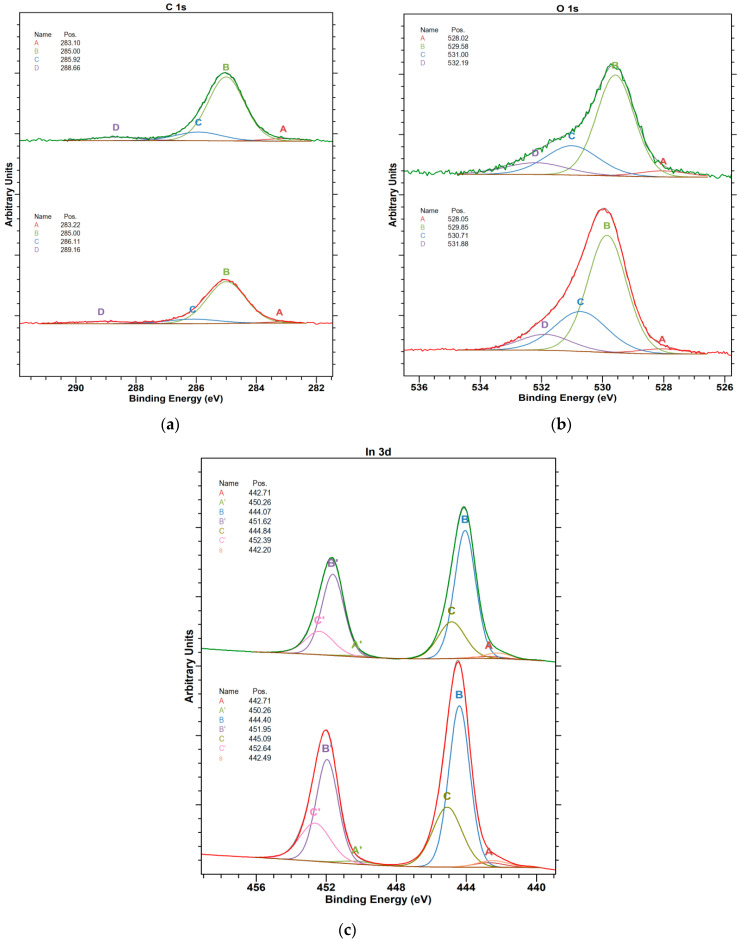
High-resolution XP spectra of In_2_O_3_layers (**a**) C 1s (A: C-metal; carbon-metal bonding, carbides, B: C-C; organic carbon, the BE suggests that it can be sp2 in majority, C: C-O; alcohol or ether groups, D: COOH, carboxylic or peptide groups), (**b**) O 1s (A: O-metal, defected oxide structure, presence of oxygen vacancies, B: O-metal, lattice oxides, C: OH, hydroxyl groups at the surface, D: O-C + H_2_O, organic compounds and water, oxygen bonded to short aliphatic chains + adsorbed water), (**c**) In 3d (A: In-C; surface carbides, B: In^3+^-O; In_2_O_3_ lattice oxide, C: In^3+^-OH; In(OH)^3^) (where: name-spectrum component (in case of doublet excitation, the maxima are depicted as A and A’, etc.).

An analysis of the high-resolution spectra for In_2_O_3_ sample (Figure 4) corroborate the conclusion on a basis of survey spectra analysis.

**Figure 5:**
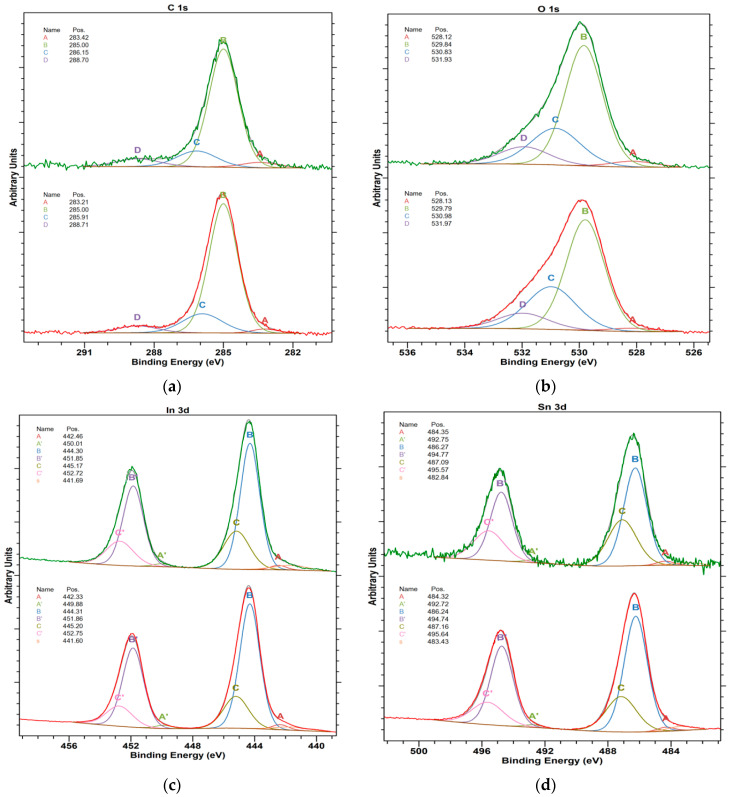
High-resolution XP spectra of ITO layers (**a**) C 1s (A: C-metal; carbon-metal bonding, carbides, B: C-C; organic carbon, the BE suggests that it can be sp2 in the majority, C: C-O; alcohol or ether groups, D: COOH, carboxylic or peptide groups), (**b**) O 1s (A: O-metal, defected oxide structure, presence of oxygen vacancies, B: O-metal, lattice oxides, C: OH, hydroxyl groups at the surface, D: O-C + H_2_O, organic compounds and water, oxygen bonded to short aliphatic chains + adsorbed water), (**c**) In three dimensions (A: In-C; surface carbides, B: In^3+^-O; In_2_O_3_ lattice oxide, C: In^3+^-OH; In(OH)_3_, (**d**) Sn 3d spectra of 25 nm ITO (bottom) and 135 nm ITO (top) sample surfaces (A: Sn-C, tin carbides, B: Sn^4+^-O; SnO_2_, C: Sn^4+^-OH; Sn(OH)_4_).

The high-resolution spectra for ITO samples are collected in Figure 5a.

The deconvolution of C 1s spectra shows four spectrum components, where the one at lowest electron binding energy (BE) of app. 283 eV is assigned to carbon–metal bonding (Figure 4a). The amount of carbon–metal bonds increases with the layer thickness. This suggests the presence of carbide species at the surface of deposited layers and supports the conclusion that these species result from the target composition, i.e., from bonding agent of In_2_O_3_ powder in the target body. The O 1s spectra acquired at both studied surfaces show the presence of oxygen vacancies (component A) (Figure 4b). The amount of vacancies increases with the layer thickness, which suggests that the vacuum conditions applied during the deposition process have an impact on vacancy formation. On the other hand, the main surface component is still a lattice oxide, as indicated by B component of O 1s spectra. Based on the analysis of In 3d (Figure 4c), where the spectrum shows three doublet components, it can be stated that the main surface component is oxide. The surfaces of In_2_O_3_ contain hydroxyl groups (component C), which is in good agreement with O 1s spectra.

The deconvolution of C 1s spectrum confirms that similar carbon species are found on both studied surfaces independently of the thickness. Similarly to C 1s, the O 1s core excitations (Figure 5b) for both studied surfaces show limited difference and presence of oxygen vacancies (component A). The amount of vacancies slightly increases with the layer thickness. The In 4d spectrum (Figure 5c) shows three doublet components, where the most intensive one is ascribed to the lattice indium oxide. The surfaces of In_2_O_3_ also contain hydroxyl groups (component C), which are in good agreement with O 1s spectra. Based on the analysis of Figure 5d for Sn 4d spectra, three electronic states of tin are found on the studied surfaces. The most intensive peak (B) can be ascribed to tin(IV) bonded to oxygen in SnO_2_ structures. The thinner film shows lower intensity of the peaks assigned to hydroxyl groups (component C), which can be well related to increasing surface roughness (surface development) with the layer thickness.

## 5. Conclusions

The material parameters of the individual layers of the electronic structure have a decisive influence on the electrical parameters of the solar cell. The material parameters include the type, thickness, and density of the anti-reflective layer electronic structure, paste composition (metallic material, organic carriers, and oxides), surface morphology, and other metallic layers, electronic structures, and distribution profile emitter admixture–dopant concentrations in the near-surface area. Technological parameters include-temperature distribution as a function of stage time drying, temperature–time distribution of the contact-making step paste-substrate, distribution of temperature as a function of time of the electronic structure cooling stage and pastes, gas composition, flow in heating zones, and sources temperature in the reactor. Obtaining a photovoltaic cell with the highest efficiency value is possible by the mutual correlation of the aforementioned parameters.

As can be seen from the measurements using the Kelvin probe, the work function Φ of In_2_O_3_ does not depend on the layer thickness, and it is at the level of 4.6 eV. This is in contrast to ITO, for which the Φ increases with layer thickness change in the range from 25 nm to 135 nm. The work function for the thickest ITO layer is 4.8 eV, which corresponds to the commercial products. AFM analysis reveals that the crystalline structure of investigated samples strongly depends on the thickness of the deposited layers. Not just the thickness, but also the optimization of doping concentration, can also influence the selection of particular grain sizes. X-ray photoelectron spectrometry (XPS) was performed to analyze the surface composition of the obtained layers. Based on the survey spectra analysis of In_2_O_3_, it was found that the sample with the thicker layer is twice more contaminated with the carbon species than a sample with a thinner layer. Based on the survey spectra analysis of ITO, it was found that both surfaces are very similar in chemical composition.

## 6. Summary

Based on the research results, it was found that:

- From the measurements using the Kelvin probe, the work function Φ of In_2_O_3_ does not depend on the layer thickness, while the work function Φ of ITO seems to depend on the thickness of the layer.
- The results of coating morphology investigations with the use of AFM show the dependence of the coating structure on thickness. It was found that thinner coatings have a structure with a smaller grain size than thicker coatings. It proves that the grains of the tested coating material grow along with the increasing thickness of the coating. Similar phenomena were explained in other works by the Ostwald-Lussac law, for example, in the work [31].
- The Kelvin probe equipped with a light source allows for testing the surface conditions of samples. For example, in photovoltaic materials, surface states play an important role in charge transfer. The depth of analysis of the investigated material is about 2 nm for the Kelvin probe and about 10 nm for XPS. The XPS method shows high sensitivity surface, which comes down to a small analytical depth, which, in the case of metals, is about 5 nm.
- Obtaining a photovoltaic cell with the highest efficiency value is possible by the mutual correlation of the material and technological parameters. The work function, valence band maximum, and electronic states in the band gap are other parameters that need to be investigated in In_2_O_3_ and ITO layers, which is also the future aim of the author’s works. We are going to discuss changes in the electronic structure induced by the defects to evaluate the applicability of band gap corrections.

## Figures and Tables

**Table 1:** Formulae and quantities characterising the tested sample’s work function [8,9,10,11].

No.	Action	Formula
1	Before the test, the determination of the reference electrode parameter ΦAu depends on the crystallographic orientation of its surface	
2	Calibration of a reference electrode with a sample from highly ordered pyrolytic graphite (HOPG)	
3	Determination of the potential difference between the gold ER and the sample.	CPD = U = (Φ_sample_ − Φ_Au_)/q
4	Determination of the charge Q accumulated on the capacitor cover.	Q = C (U-V), after differentiation dQ/dt = dC/dt (U-V *)
5	Designation of the work function sought for the sample	Φ_sample_ = Φ_Au_ + qV eV

Where: ΦAu—the value is 5.31–5.47 eV, Φ HOPG—the work function value is 4.40–4.67 eV, q-elementary charge 1.602 × 10^−19^ C, C—the sample is unknown and periodically dependent on time, dC/dt value is unknown and periodic in time, * the expression in parentheses disappears, so the equation is satisfied.

**Table 2:** List of published publications on general topics by three databases [22,23,24].

The Issue You Are Looking For	WOS	SCOPUS	ELSEVIER
Kelvin probe	6.801	6.295	1
Kelvin probe + Contact potential difference	627	532	9.031
Kelvin probe + Transparent Conductive Oxides	27	21	1.029
Contact potential difference + Transparent Conductive Oxides	21	7	13.952

**Table 3:** List of published publications according to two databases, taking into account specific issues [23,24].

WOS	SCOPUS	WOS	SCOPUS	WOS	SCOPUS
AZO and In_2_O_3_		FTO and In_2_O_3_		In_2_O_3_	
6	6	38	20	9.116	5.710
AZO and SnO_2_		FTO and SnO_2_		SnO_2_	
22	36	1478	401	34.258	25.305
ITO		Kelvin probe and ITO		work function and ITO	
25.43	10.15	89	185	1.285	1.72
Kelvin probe and In_2_O_3_		TCO and In_2_O_3_		work function and In_2_O_3_	
20	9	561	376	310	137
Kelvin probe and SnO_2_		TCO and SnO_2_		work function and SnO_2_	
66	43	579	516	862	424

**Table 4:** The results of the work function, thickness, and roughness of the investigated samples [7,8,9,10].

The Name of The Oxides	The Thickness of Layers [nm]	The Work Function of Layers [eV]	Roughness R_a_ [nm]
In_2_O_3_	15	4.6	0.1
	25	4.6	0.2
	45	4.7	0.5
	65	4.6	0.9
	135	4.7	1.2
ITO	Ref	4.9	1
	15	4.6	0.2
	25	4.3	0.2
	45	4.4	0.2
	65	4.7	1
	135	4.8	1

**Table 5:** The results of survey spectra of In_2_O_3_ and ITO sample surfaces samples.

In_2_O_3_	25 nm	135 nm
Name *	At%	At%
C 1s	18.4	32.6
O 1s	38.0	33.1
In 3d	43.5	34.2
**ITO**	25 nm	135 nm
Name *	At%	At%
C 1s	24.8	20.9
O 1s	37.9	38.0
In 3d	34.2	37.6
Sn 3d	2.9	3.3

(Where: name *-core excitation for given element, At%-atomic concentration at the surface layer).

## Data Availability

Not applicable.
